# An Isolated Medial Patellofemoral Ligament Reconstruction with Patellar Tendon Autograft

**DOI:** 10.1155/2013/637678

**Published:** 2013-10-13

**Authors:** Dariusz Witoński, Rafał Kęska, Marek Synder, Marcin Sibiński

**Affiliations:** ^1^Department of Reconstructive Surgery and Arthroscopy of the Knee, Medical University of Łódź, 91-002 Łódź, 75 Drewnowska, Poland; ^2^Clinic of Orthopaedics and Paediatric Orthopaedic, Medical University of Łódź, 91-002 Łódź, 75 Drewnowska, Poland

## Abstract

The aim of the study was to evaluate the results of the medial patellofemoral ligament reconstruction with a medial strip of patellar tendon autograft after a minimum 2-year followup. Ten patients (10 knees) were operated on by one surgeon, according to the modified technique, described by Camanho, without any bone plug at free graft end. The mean age of the patients was 27.2 years (ranging from 18 to 42 years). The mean follow-up period was 3 years and 7 months. All patients were reviewed prospectively. At the last follow-up visit, all the patients demonstrated a significant improvement in terms of patellofemoral joint stability, all aspects of the KOOS questionnaire, and Kujala et al.'s score (59.7 points preoperatively and 84.4 points at the last followup). No patient revealed recurrent dislocation. The SF-36 score revealed a significant improvement in bodily pain, general health, physical role functioning, social role functioning, and physical functioning domains. The described MPFL reconstruction with the use of the medial 1/3rd of patella tendon is an effective procedure that gives satisfactorily patellofemoral joint functions, improves the quality of life, and provides much pain relief. It is relatively simple, surgically not extensive, and economically cost-effective procedure.

## 1. Introduction 

Traumatic lateral dislocation of the patellofemoral joint leads to the rupture of the medial patellofemoral ligament, usually in the femoral attachment side [1, 2]. Clinical observations and bibliographic data suggest that surgical treatment does not statistically significantly reduce the incidence of recurrent dislocation [3, 4]. However, in case of posttraumatic recurrent instability, the reconstruction of the medial patellofemoral ligament (MPFLR) remains the procedure of choice. The literature has described several procedures which used different periarticular structure as graft material for reconstruction. In the year 2007, Camanho et al. described a novel technique of medial patellofemoral reconstruction, using a patellar tendon graft [4]. Since April 2008 we have started to use a modified version of this technique at our Department and, in 2010, our preliminary report was published [5]. Our early results were very promising in terms of patellofemoral joint functionality, the quality of life, sport activity, or pain sensation. Several advantages of the procedure were noted. No bone tunnels were necessary in the patella, only one screw anchor was used, and no tubercle bony attachment was involved [5]. As results of the procedure are very satisfactory, we have still been using it in our everyday practice. The number of patients has increased and follow-up periods are longer. To our best knowledge, there has been no previous attempt to analyze a medium-term followup of MPFLR, using this particular source of the graft.

The aim of the study is to evaluate the results of medial patellofemoral ligament reconstruction with a medial strip of patellar tendon autograft after minimum 2-year followup. We hypothesized that this procedure is equal with other graft sources used for MPFLR, improving patellofemoral joint function and quality of life and giving pain relief. 

## 2. Materials and Methods 

Since April 2008 till 2012, nineteen patients with unilateral, posttraumatic, chronic, and lateral patellofemoral joint instability had been treated at our Department with MPFL reconstruction, using an autologous patellar tendon graft. All patients were reviewed prospectively. The follow-up period was longer than 2 years for 11 of those patients. Ten of them were available for final followup. The characteristics of patients are shown in Table 1. The mean follow-up period was 3 years and 7 months, ranging from 2 years to 4 years and 7 months. The one missing patient did not respond to our invitation to the hospital outpatient clinic. The inclusion criterion was history of traumatic patellar luxation as a starting point of recurrent lateral patellar instability. All patients underwent complete clinical examination of the lower extremity and routine examination of the knee as well as the patellofemoral joint. Exclusion criteria were any abnormalities of pelvic geometry, femoral anteversion, abnormal *Q* angle, trochlear dysplasia, patella baja or alta, external tibial torsion, or hindfoot position as well as generalized laxity.

Follow-up visits were performed after 1, 3, 6, 12, and 24 months from operation and at the endpoint of followup. During those visits patient symptoms were recorded, including the incidents of patella dislocation/subluxation. The following several assessment methods were used for evaluation: Kujala scale, Knee Injury and Osteoarthritis Outcome Study (KOOS) questioner, and SF-36 questioner version 2 (Short-Form 36 v.2) (license number H1-031207-30347) [6–8]. 

The patients were operated on by one surgeon, according to the technique, described by Camanho et al. [4], without any bone plug at free graft end. The operations were performed under subarachnoid anaesthesia, in bloodless operative field, using a pneumatic tourniquet. In all the cases, the extra-articular part of ligament reconstruction was preceded by knee arthroscopy to determine the scope and extent of potential damage to the articular cartilage. Skin incision started from the proximal part at the level of the upper edge of the patella, located midway between the medial epicondyle of the femur and the medial edge of the patella, directing them towards the area of upper-medial tibial tuberosity. After visualization of the patellar tendon, its medial-third part was separated from the tuberosity of the tibia, first distally and then proximally. The graft was left attached to upper-medial quadrant of the patella. The free end of the graft was sewn by Krackow's stich. After unveiling the medial epicondyle of the femur, the knee was flexed to about 30–40° angle and, using the “anchor” of titanium, the distal end of the graft was stabilized proximally and slightly backward from the medial epicondyle of the femur, between the epicondyle and the great adductor tubercle. The location of femoral attachment of the reconstructed ligament was radiologically controlled. The graft was sewn to the vastus medialis muscle with several sutures, providing a dynamic element of stability. We did not do “lateral patellar release,” due to the unrestricted passive medial patellar translation, keeping in mind that the lateral release may potentially increase the lateral patellar side instability [9]. 

The wound was closed in layers with continuous sutures. No drainage was installed. Intradermal sutures were applied by the standard technique. The tourniquet was released after applying a sterile dressing and a Jones soft padded bandage.

The knee joint was immobilized in a hinged brace with the range of motion between 0° and 90°. Rehabilitation was initiated on the first postoperative day and the management was identical in all the patients. On the first day after surgery, the patients were verticalized and they exercised passive full extension, as well as active flexion of the operated knee to the angle of 90°, with continuous passive motion splint (CPM splint) as a postoperative therapy. Straight leg raising exercises were recommended and walking with elbow crutches with weight bearing of the operated limb “as tolerable.” The patients gradually introduced closed kinetic chain, as well as proprioceptive exercises. In 3-4 weeks after surgery the hinged brace was unlocked for the full range of motion. After full range of motion was obtained, strengthening exercises were added. Return to sport or recreational activity was allowed after 3 months from the operation, depending on muscle strength of the operated limb.

Statistical analysis was performed with the Statgraphics Plus software for Windows v. 5.1. Normal distribution was checked with the Shapiro-Wilk test. A comparison between preoperative and postoperative results was performed with Wilcoxon Rank Tests and Student *t-*test for dependent samples. *P* values below 0.05 were considered clinically significant. 

## 3. Results 

**Figure 1:**
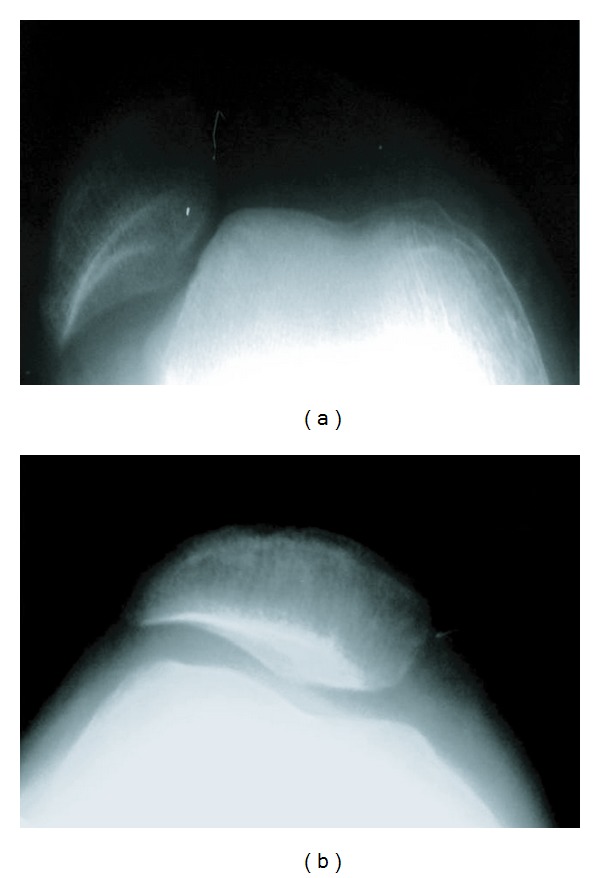
Skyline view radiographs show patella location (a) before and (b) after surgery.

At the last followup, all the patients demonstrated a significant improvement in terms of patellofemoral joint stability, compared to the preoperative status. No recurrent dislocation was identified in any of the patients nor any deterioration of knee function (Figure 1). Significantly improved results were found, regarding Kujala et al.'s classification, all the aspects of the KOOS questionnaire, and most aspects of the SF-36 questionnaire. A more detailed information, including scores in the subgroups, is presented in Table 2.

## 4. Discussion 

 Many variants of MPFL reconstruction have been described during the last years, which technique is still widely accepted to restore posttraumatic patellofemoral stability. Some surgeons use allografts or artificial ligament, while others prefer autografts. For autologous repairs, hamstring tendons, quadriceps or adductor magnus tendons are in use [10–18]. 

Since 2008, we have been using the medial part of patella tendon, according to the modified method of Camanho [4] without any bone plug at free graft end. The treatment outcomes, obtained in our patients, are similar to the literature results of other operative methods. In the described group of patients, the patellofemoral joint function significantly improved by 25 points, according to Kujala et al.'s classification after, at least, 2-year followup. The patients reported also significant improvement in all the aspects of the KOOS questionnaire. The SF-36 scores revealed improvements, regarding pain sensation, general health status, and in physical and social domains. 

A comparison among results, reported in the literature, might be difficult as they originate from different classification systems although the mean improvement from 23 to 42 points, according to Kujala et al., could be expected, when comparing pre- and postoperative status [13, 15, 16, 19–21]. It is worth mentioning that, before operation, the knee function by this classification system was almost 60 among our patients, being higher than in most of the published studies [13, 15, 16, 19]; thus, the improvement by 25 points seems to be a fairly good outcome. Many of those publications do not mention the status of patellofemoral joint in terms of osteoarthritis. In our opinion, this information is important when comparing results from different studies.

Schöttle et al. and Kumar et al., while using semitendinosus tendon graft, noted improvement from 55 to 85.7 and from 46.9 to 89 points, according to Kujala's classification, respectively [13, 16]. Howells et al. reported a prospective analysis in 219 patients, using an autologous semitendinosus graft. A significant improvement was noted in 193 patients. Females with atraumatic recurrent dislocation and patients with a history of previous surgeries had worse results [22]. Goorens et al. and Christiansen et al. used autologous gracilis tendon grafts for MPFL repair [15, 18]. In the study of Christiansen et al., the Kujala's score changed from 46 to 84 points [15]. Sillanpää et al. used adductor magnus muscle tendon and noted improvement to 88 points [19]. Nomura et al. reported 91% of excellent and good results with the use of artificial ligament for MPFL reconstruction in a midterm followup [23]. 

More recent studies show significant improvements and favorable results after “more anatomical” patella double tunnel reconstruction [14, 17, 21]. The improvement in Kujala scores ranged from 23 to 41 in those articles and seems to be similar to the results of other operative technics [13, 15, 16, 19, 20]. Definitely, the double tunnel operation is more extensive to simple, single tendon or patellar tendon reconstruction.

 Despite the fact that our operative protocol allows to achieve similar outcomes, compared to other technics, it seems to have several other advantages. There is no need to harvest semitendinosus or gracilis tendon. Medial hamstring muscles, which protect the anterior cruciate ligament, remain intact [24, 25]. No tunnels are performed within the patella, what may prevent additional complications, like patellar fracture [15, 26]. As only one titanium anchor is applied on the femoral, this procedure is relatively cost-effective. Furthermore, the use of many implants to stabilize the graft increases the risk of pain and local inflammation around anchors or screws. The need of reoperation to remove the stabilizing implant may be as high as 7–10% [15, 20]. 

 Intraoperative evaluations showed that the patellar tendon, together with its part, covering tibial tuberosity, is long enough as a graft in MPFL reconstruction. This has convinced us to modify the operative technique, described by Camanho et al. They used the medial 1/3rd of the patella tendon, together with a bone graft of tibial tuberosity [4]. We believe that limiting the procedure to soft tissue graft only reduces pain sensation at donor's side and allows for a more aggressive rehabilitation protocol. 

The strengths of our study include prospective evaluation as well as standardization of hospitalization procedures with participation of the same operating team and the use of identical rehabilitation regimens. It has also been established that both the functional status and the quality of life can be improved, as reported by the patients themselves, rather than speculated by physicians from clinical examinations (assessment with patient-related outcomes (PROs)). However, since different PROs have different abilities to capture real symptoms and disabilities not only experienced by but also important for patients, we also used different and joint specific questionnaires. 

The study has some limitations. The number of subjects is not informative enough to assess the strength of obtained results, being, however, sufficient for a preliminary evaluation, while providing foundations and a good starting point for further analyses.

## 5. Conclusions

The described MPFL reconstruction with the use of the medial 1/3rd of patella tendon is an effective procedure in patient with posttraumatic patellofemoral instability that gives satisfactory results, regarding the patellofemoral joint function, improves quality of life, and provides pain relief. The proximal graft attachment to the patella is left and only one anchor suture is needed at the femoral side. The procedure is relatively simple, surgically not extensive and economically cost-effective, while eliminating any needs for tibial tuberosity grafts, patella tunnels, or hamstring tendon damage as compared to other current used techniques.

## Figures and Tables

**Table 1:** Patients' demographics.

Characteristic	
Number (number of men)	10 (4)
Age, mean (SD)	18–42, 27.2 (8.1) years
Number of dislocations	
Less than 5	1
More than 5	9
Duration of symptoms, mean (SD) years	2–25, 11.4 (7.6)
Associated damage	
Chondromalacia	6
OA	4

**Table 2:** Kujala, KOOS, and SF-36 subscale scores of 10 subjects with MPFL reconstruction after, at least, a 2-year followup.

	Before operation			Last followup			*P* value
	Min	Max	Mean	Min	Max	Mean	
Kujala et al.'s classification [6]	11.0	81.0	59.7	56.0	98.0	84.4	**0.02**
KOOS [7]							
Pain	27.8	94.4	75.8	77.8	100	92	**0.04**
Symptoms	25.0	92.8	64.3	53.6	100	83.2	**0.04**
Function, daily functions	22.1	100	82.4	89.7	100	95.4	**0.04**
Functions, sports, and recreational activities	0	80.0	43.5	15.0	100	79.0	**0.03**
Quality of life	0	62.5	27.5	37.5	93.8	66.3	**0.0004**
SF-36 [8]							
Physical functioning	21.3	52.8	42.5	42.3	57.0	53.0	**0.01**
Physical role functioning	17.7	56.9	45.9	52.9	56.9	55.4	**0.008**
Bodily pain	19.9	62.1	44.3	46.1	62.1	55.2	**0.03**
General health	32.9	60.1	47.0	45.8	63.9	54.8	**0.03**
Vitality	27.1	58.3	47.4	36.5	67.7	54.3	0.2
Social role functioning	13.2	56.8	41.6	45.9	56.8	53.3	**0.03**
Emotional role functioning	9.2	55.9	48.5	40.3	55.9	51.6	0.6
Mental health	21.8	55.6	44.7	33.1	55.6	47.2	0.7

Bold font refers to the value of statistical significance.

## References

[1] Nomura E (1999). Classification of lesions of the medial patello-femoral ligament in patellar dislocation. *International Orthopaedics*.

[2] Nikku R, Nietosvaara Y, Aalto K, Kallio PE (2005). Operative treatment of primary patellar dislocation does not improve medium-term outcome: a 7-year follow-up report and risk analysis of 127 randomized patients. *Acta Orthopaedica*.

[3] Mikashima Y, Kimura M, Kobayashi Y, Miyawaki M, Tomatsu T (2006). Clinical results of isolated reconstruction of the medial patellofemoral ligament for recurrent dislocation and subluxation of the patella. *Acta Orthopaedica Belgica*.

[4] Camanho GL, Bitar AC, Hernandez AJ, Olivi R (2007). Medial patellofemoral ligament reconstruction: a novel technique using the patellar ligament. *Arthroscopy*.

[5] Witońki D, Keska R, Bira M (2010). Reconstruction of the medial patellofemoral ligament with patellar tendon autograft—preliminary report. *Chirurgia Narzadów Ruchu i Ortopedia Polska*.

[6] Kujala UM, Jaakkola LH, Koskinen SK, Taimela S, Hurme M, Nelimarkka O (1993). Scoring of patellofemoral disorders. *Arthroscopy*.

[7] Roos EM, Lohmander LS (2003). The Knee Injury and Osteoarthritis Outcome Score (KOOS): from joint injury to osteoarthritis. *Health Qual Life Outcomes*.

[8] Patel AA, Donegan D, Albert T (2007). The 36-Ltem short form. *Journal of the American Academy of Orthopaedic Surgeons*.

[9] Christoforakis J, Bull AMJ, Strachan RK, Shymkiw R, Senavongse W, Amis AA (2006). Effects of lateral retinacular release on the lateral stability of the patella. *Knee Surgery, Sports Traumatology, Arthroscopy*.

[10] Noyes FR, Albright JC (2006). Reconstruction of the medial patellofemoral ligament with autologous quadriceps tendon. *Arthroscopy*.

[11] Sillanpää PJ, Mäenpää HM, Mattila VM, Visuri T, Pihlajamäki H (2009). A mini-invasive adductor magnus tendon transfer technique for medial patellofemoral ligament reconstruction: a technical note. *Knee Surgery, Sports Traumatology, Arthroscopy*.

[12] Teitge RA, Torga-Spak R (2004). Medial patellofemoral ligament reconstruction. *Orthopedics*.

[13] Schöttle PB, Fucentese SF, Romero J (2005). Clinical and radiological outcome of medial patellofemoral ligament reconstruction with a semitendinosus autograft for patella instability. *Knee Surgery, Sports Traumatology, Arthroscopy*.

[14] Toritsuka Y, Amano H, Mae T (2011). Dual tunnel medial patellofemoral ligament reconstruction for patients with patellar dislocation using a semitendinosus tendon autograft. *Knee*.

[15] Christiansen SE, Jacobsen BW, Lund B, Lind M (2008). Reconstruction of the medial patellofemoral ligament with gracilis tendon autograft in transverse patellar drill holes. *Arthroscopy*.

[16] Kumar V, Panagopoulos A, Triantafyllopoulos JK, Niekerk van L (2008). Medial patello-femoral ligament reconstruction with semitendinosus re-routing for the treatment of traumatic patella dislocation. *Journal of Bone & Joint Surgery*.

[17] Han H, Xia Y, Yun X, Wu M (2011). Anatomical transverse patella double tunnel reconstruction of medial patellofemoral ligament with a hamstring tendon autograft for recurrent patellar dislocation. *Archives of Orthopaedic and Trauma Surgery*.

[18] Goorens CK, Robun H, Hendrickx B, Delport H, Mulder KD, Hens J (2010). Reconstruction of the medial patellofemoral ligament for patellar instabifity using an autologous gracilis tendon graft. *Acta Orthopaedica Belgica*.

[19] Sillanpää P, Mattila VM, Visuri T, Mäenpää H, Pihlajamäki H (2008). Ligament reconstruction versus distal realignment for patellar dislocation. *Clinical Orthopaedics and Related Research*.

[20] Steiner TM, Torga-Spak R, Teitge RA (2006). Medial patellofemoral ligament reconstruction in patients with lateral patellar instability and trochlear dysplasia. *American Journal of Sports Medicine*.

[21] Zhang L, Li Z, Liu J, Sun J, Ma J (2010). Anatomical double bundle reconstruction of medial patellofemoral ligament with allograft tendon in patellar dislocations. *Chinese Journal of Reparative and Reconstructive Surgery*.

[22] Howells NR, Barnett AJ, Ahearn N, Ansari A, Eldridge JD (2012). Medial patellofemoral ligament reconstruction: a prospective outcome assessment of a large single centre series. *The Bone & Joint Journal*.

[23] Nomura E, Horiuchi Y, Kihara M (2000). A mid-term follow-up of medial patellofemoral ligament reconstruction using an artificial ligament for recurrent patellar dislocation. *Knee*.

[24] Myer GD, Ford KR, Barber Foss KD, Liu C, Nick TG, Hewett TE (2009). The relationship of hamstrings and quadriceps strength to anterior cruciate ligament injury in female athletes. *Clinical Journal of Sport Medicine*.

[25] Hewett TE, Myer GD, Ford KR (2005). Biomechanical measures of neuromuscular control and valgus loading of the knee predict anterior cruciate ligament injury risk in female athletes: a prospective study. *American Journal of Sports Medicine*.

[26] Thaunat M, Erasmus PJ (2008). Recurrent patellar dislocation after medial patellofemoral ligament reconstruction. *Knee Surgery, Sports Traumatology, Arthroscopy*.

